# “Thinking on your feet”—a qualitative study of debriefing practice

**DOI:** 10.1186/s41077-016-0011-4

**Published:** 2016-04-02

**Authors:** Kristian Krogh, Margaret Bearman, Debra Nestel

**Affiliations:** 1 Centre for Health Sciences Education, Aarhus University, 8200 Aarhus N Aarhus, Denmark; 2 HealthPEER, Faculty of Medicine, Nursing and Health Sciences, Monash University, Victoria, Australia; 3 School of Rural Health, HealthPEER, Faculty of Medicine, Nursing and Health Sciences, Monash University, Victoria, Australia

**Keywords:** Debriefing, Simulation-based education, Blended approach to debriefing, Faculty development

## Abstract

**Background:**

Debriefing is a significant component of simulation-based education (SBE). Regardless of how and where immersive simulation is used to support learning, debriefing has a critical role to optimise learning outcomes. Although the literature describes different debriefing methods and approaches that constitute effective debriefing, there are discrepancies as to what is actually practised and how experts or experienced debriefers perceive and approach debriefing. This study sought to explore the self-reported practices of expert debriefers.

**Methods:**

We used a qualitative approach to explore experts’ debriefing practices. Peer-nominated expert debriefers who use immersive manikin-based simulations were identified in the healthcare simulation community across Australia. Twenty-four expert debriefers were purposively sampled to participate in semi-structured telephone interviews lasting 45–90 min. Interviews were transcribed and independently analysed using inductive thematic analysis.

**Results:**

Codes emerging through the data analysis clustered into four major categories: (1) **Values**: ideas and beliefs representing the fundamental principles that underpinned interviewees’ debriefing practices. (2) **Artistry**: debriefing practices which are dynamic and creative. (3) **Techniques**: the specific methods used by interviewees to promote a productive and safe learning environment. (4) **Development**: changes in interviewees’ debriefing practices over time.

**Conclusions:**

The “practice development triangle” inspired by the work of Handal and Lauvas offers a framework for our themes. A feature of the triangle is that the **values** of expert debriefers provide a foundation for associated **artistry** and **techniques**. This framework may provide a different emphasis for courses and programmes designed to support debriefing practices where microskill development is often privileged, especially those microskills associated with **techniques** (plan of action, creating a safe environment, managing learning objectives, promoting learner reflection and co-debriefing). Across the levels in the practice development triangle, the importance of continuing professional development is acknowledged. Strengths and limitations of the study are noted.

## Background

Health professional education has witnessed a significant increase in the use of simulation since in the 1980s. What was once an area of interest for a limited group of clinical educators is now fully integrated into many health professional curricula [1–3]. There is a wide spectrum of simulators supporting the development of teaching very simple skills, such as injection of fluid into an orange, as well as the very complex, interprofessional teamwork in immersive scenarios using highly technological manikins. The latter type of simulation allows for optimising interactions between people, tasks and organisational conditions [4, 5].

Immersive simulations [6] generally include a debriefing where the facilitator leads a group discussion that reviews the simulation experience and provides feedback to the participants. Debriefing is defined by Cheng et al. “as a discussion between two or more individuals in which aspects of a performance are explored and analysed with the aim of gaining insights that impact the quality of future clinical practice” [7]. The simulation-based education (SBE) literature highlights the role of debriefing in participants’ learning [1, 2, 8–12]. A consistent theme is that debriefing is important in promoting integration of participants’ experiences through reflection, which is likely to improve clinical practice [8, 9, 12]. The literature also outlines many approaches to debriefing [13–21].

Cheng et al.’s [7] systematic review on debriefing in technology-enhanced simulation indicates the broad range of literature available regarding debriefing, particularly the many models and approaches for debriefing—some are context specific while others are generic. In the same year, Waznonis [22] published a literature review on evaluations for simulation-related debriefings in educational settings and identified 22 methods and seven evaluations, and more have been reported since this review [20, 23]. In general, the literature describes how debriefing could or should be done to gain the most from the preceding experiential learning activity. However, there is little evidence supporting one method over another. It is likely that several factors are important such as what is taught, the level of learners, their previous experiences, the individual facilitator’s background and the learning environment [13, 17, 24]. These and other debriefing characteristics are often incompletely reported [7]. The actual use of these models by expert debriefers is rarely mentioned in studies on impact of SBE and if so, only briefly [25]. Although the literature describes how debriefing should be conducted, there is less information about how debriefings actually take place.

Closely related to debriefing in SBE is the notion of the pre-simulation briefing (synonymous with the terms introduction, orientation and pre-briefing). The role of the pre-simulation establishing a safe learning environment is acknowledged in the literature [26]. While important, this study is focused on debriefing; we acknowledge the significant role of the briefing and value it for what it does rather than exploring how it is done.

In summary, the literature provides extensive and valuable guidance on how to approach debriefing as well as an understanding of the associated role of briefing. What is less well known is how educators debrief in actual teaching environments, with significant and occasional challenges such as limited time, disinterested learners and failing technology. In seeking to understand how to optimise debriefing “on the ground”, we aim to understand how experts approach their debriefing practices.

Our research question is what are the debriefing practices of expert debriefers after immersive manikin-based simulations?

## Methods

This study used a qualitative approach to explore debriefing in immersive scenario- and manikin-based simulation. With the researcher as an active interpreter, data was analysed inductively, with a continued awareness of researchers’ own preconceptions and backgrounds [27]. The three authors have extensive experience with SBE and different debriefing approaches in a variety of contexts and simulation modalities. KK has a medical background while MB and DN are both experienced health professional and simulation educators with extensive experience conducting qualitative research. An additional focus on the study, which emerged as a separate theme during analysis, was the practical uses of video-assisted debriefing. This narrow focus analysis is described elsewhere [28]. The analysis of data presented in this study has not been reported previously.

### Ethics

Ethical approval was obtained from Monash University Human Research Ethics Committee, Australia—Project Number: CF12/1604 – 2012000867.

### Sampling and data collection

The participants sampled for this study were purposively selected from 66 peer-nominated expert debriefers in immersive manikin-based simulations. Senior faculty (*n* = 13) of a national faculty development programme for simulation educators (AusSETT) funded by the Australian government-nominated expert debriefers for inclusion in the study [29–31]. The nominating senior faculty are established and respected simulation educators, many of whom have served with the Australian main association (or “peak body”) for healthcare simulation and therefore have a wide reach across the healthcare simulation community [29].

Selection criteria for the purposive sampling from the 66 nominations were (1) multiple nominations, (2) diversity of affiliation (simulation centre, hospital and university affiliation) and (3) diversity of clinical and educational background. Additionally, employment location was included as a criterion, with a view to seek representation across all Australian states and territories. The data pertaining to these criteria were provided when known by the nominators or retrieved through internet search. Relevant demographic data was collected and checked during the interviews.

Thirty potential respondents purposively selected were contacted by email. Five declined, were unable to participate or did not respond, and one interviewee was excluded after the interview due to lack of debriefing experience (less than 4 years). With a focus on immersive manikin-based simulations, all respondents were explicitly asked to consider and discuss debriefing relevant to this modality. One interviewer (KK) conducted the 24 individual semi-structured exploratory telephone interviews lasting 45–90 min (a total of 25 h 38 min). Interviews followed a semi-structured topic guide that was piloted and adjusted prior to use (see Appendix). The topic guide was based on literature and designed to address our research question. Interviews were audio recorded and transcribed (a total of 470 pages) by a professional transcriber. The interviewer (KK) then read and listened to all interviews to verify accuracy.

The 24 debriefers (10 women and 14 men) whose interview transcripts were included in the analysis had between 4 and 23 years of debriefing experience (mean 9.7 years). Thirteen described themselves as full-time educators and 11 as part-time, and they came from a variety of disciplines including medicine, nursing, midwifery, paramedicine and allied health in 20 different institutions/organisations across Australia. Eight debriefers reported mainly facilitating training for students, 16 for graduates in a range of simulation types from skill and algorithm training to immersive full-scale simulation, both in situ and/or within a simulation facility.

### Analysis

The first step in the analysis was to develop a coding framework. The authors (KK, MB and DN) inductively and independently coded randomly selected transcripts (*n* = 12; KK = 5, MB = 3 and DN = 4). Through this process, we jointly rearranged and renamed codes, and developed a framework of higher order themes by consensus, in the qualitative description tradition [32]. That is, analysis closely represented what the participants themselves described. All 24 transcripts were then coded according to the framework using interpretive thematic analysis [33, 34], by a single researcher (KK). This paper deals with the higher order themes of “practice” and “development of expertise”.

The second step of analysis was to consider these higher order themes in more depth. The authors (KK, MB and DN) inductively re-analysed eight purposively selected interviews (four each with two overlapping,) using a more interpretive lens [27]. A total of 36 codes emerged during this stage, reaching saturation after approximately 16 interviews.

The third step was to create a summary of each code with descriptive recurrent quotes (KK). That is, we returned to the transcripts to identify text for each code seeking confirming and disconfirming evidence. These summaries of descriptive recurrent quotes were reviewed and thematised (KK and MB) resulting in four categories. At this point, we also reduced the 36 codes to 18 themes and 10 subthemes (Table 1). The reduction was made possible because of the overlapping codes.

**Table 1:** Overview of the emerging categories, themes and subthemes

**Category**	**Themes**	**Subthemes**
**Values**	Philosophies	*Learner-centredness*
	Theories	
	Impact	
**Artistry**	Thinking on your feet	*Flexibility*
		*Balancing*
		*Prioritising*
	Blended debriefing	
	Strategizing	
	Personal touch	*Frame*
		*Honesty*
		*Stance*
**Techniques**	Plan of action	*Structuring*
		*Methods*
		*Sequencing*
	Creating a “safe” learning environment	
	Managing learning objectives	
	Promoting learner reflection	
	Co-debriefing	
**Development**	Transformation	
	Challenges	
	Becoming comfortable with the uncomfortable	
	Care about their practice	
	Learning from and with peers	
	Training	

The fonts used for Categories, Themes and Subthemes in Table 1 are corresponding to those used throughout the article

The fourth step consisted of a critical review of the data against the categories, themes and subthemes (KK, MB and DN). This final step ensures that the findings are truly representative of the data.

NVivo 10 (NVivo version 10.0.638.0, QSR International) was used to manage data. An audit trail of analytic moves was maintained.

## Results

The data analysis clustered into four major categories: **values**, **artistry**, **techniques** and **development** (Table 1: overview of the emerging categories, themes and subthemes). These represent the totality of the debriefing practices described by the interviewees. These categories and their component themes and subthemes are described in the rest of this section, illustrated with representative quotes.

### Values

The interviewees explicitly and implicitly expressed core sets of ideas and beliefs that represented the fundamental principles that underpinned their debriefing practices. Three themes emerged philosophies, theories and impact. Interviewee’s **values** appeared relatively stable.

#### Philosophies

Most of the interviewees expressed a philosophy of debriefing; the debriefer is a facilitator, who must be dedicated, honest, genuinely curious and possess the abilities to facilitate a reflective debriefing.

*“The idea of being an advocate and genuinely curious I think is fundamental to good debriefing.” Interviewee #13*

*Learner-centredness* was a subtheme. This notion, of following the learners’ needs and objectives, was a common philosophy underpinning debriefing approaches.

#### Theories

Although few interviewees described explicit educational theories or theorists, there was an overwhelming constructivist influence in their practices. That is, the interviewees noted the importance of acknowledging the participants’ prior experiences and ideas, and their thoughts and feelings about the simulation activity [35]. Constructivism was also manifested in their talk about *scaffolding* and *experiential learning* [36, 37]. Theorists that were cited included Kirkpatrick [38], Kolb [39] and Lave and Wenger [40]. Some interviewees identified educationalists who had shaped their thinking about simulation and particularly their debriefing.

*“I think whether you label someone an educational theorist or not, whether they've contributed something that contributes to education theory then that's important…. So lots and lots of people have contributed really important aspects that make up one big picture I think.” Interviewee #4*

The interviewees described themselves as “doers” rather than “academics”; and there was an impression of regret as some interviewees expressed the desire to be more academically grounded.

#### Impact

There was a sense among interviewees that the debriefing creates an opportunity for all learners to adjust their perceptions of the world and reinforce, improve or change practice. In simulation, changed and improved practice is often displayed after the consecutive scenarios. Although there was perceived value in learners improving in simulation, the overall objective was to improve clinical practice.

*“Pull out the salient bits that should be transferred to the real clinical environments because we really don't want people to be getting better at simulation, we want people to be getting better at clinical practice” Interviewee #4*

Improvements that are promoted and reinforced through debriefing may potentially improve clinical practice.

### Artistry

**Artistry** refers to the “creative skill or ability” [41] noted in the interviewee’s descriptions of their practice. The themes within this category distinguish these interviewees as experts. The themes draw heavily from each other: “thinking on your feet” is required to blend models and manage learning objectives, and is also heavily informed by the personal characteristics of the debriefer.

#### Thinking on your feet

The first, and in some ways, the most significant theme of the artistry category, was the importance of being able to facilitate in a dynamic environment and “think on your feet” (Interviewee #12). This varied somewhat dependent on the learners’ level and the context. Across the three subthemes’ *flexibility*, *balancing*, and *prioritising*, there is an important idea of constant vigilance as debriefings may constantly change depending on learners’ interactions and reactions.

*“Debriefings are very reactive. You have to be watching what’s happening as you’re going and modifying what you’re doing” Interviewee #17*

*Flexibility* revolved around the notion that interviewees had a repertoire of debriefing methods on which to draw and made in-the-moment decisions to select the approach that best suited the current situation. This often led to a blended debriefing approach, which is expanded in the next theme.

*Balancing* and *prioritising* include managing the various different agendas inherent within a simulation.

#### Blended debriefing

A consistent theme was the interviewees’ blending of debriefing approaches [20], that is, the use of different models and techniques to approach debriefing.

*“My personal approach is more of a blended approach I have to say. I know of all of the different styles of debriefing. I’ve found that no one style seems to fit me for all of the types of debrief that I do.” Interviewee #5*

The interviewees’ blending of debriefing models and techniques was dynamic and dependent upon personal preferences, the learners’ perceived needs, and the scenario as experienced by the debriefer. This complex practice was therefore regarded more as artistry than as a technical process.

*“Depending on what the simulation is and depending on what the participants are like when they enter the debriefing room, use all the different tools that are available to try and create your style for that particular debrief” Interviewee #16*

#### Strategizing

The interviewees described having different strategies depending on the level of the learner and type of simulation or course. Strategizing was dynamic, as the way the scenario unfolds and the learners’ perception of the scenario may change the initial strategy. This counterbalanced *thinking on your feet* — it suggested that interviewees did not start the debrief de novo, but had a range of ways in which they could achieve their agenda. These strategies could include various techniques, as outlined below.

#### Personal touch

Artistry is reliant on the artist; this theme encapsulates the unique and personal approach that defines debriefing as a creative practice. Three subthemes emerged: *frame*, *honesty*, and *stance*. *Frame* relates to the interviewees’ background, preconceptions and experiences.

*“… I think a background in emergency medicine is helpful from that perspective because I don't expect prescription for everything I see. I mean I've never had another background but I’m used to unexpected things and it doesn’t faze me.” Interviewee #12*

*Honesty* enables a constructive discussion, but simultaneously, there was a sense that the presentation of honesty was dependent on the debriefer:

*“I personally think that learners don’t value pussyfooting around tricky situations or tricky questions or answers. I think they just want to hear sometimes from the instructor what your view is” Interviewee #1*

Debriefers also declared their *stance*, that is, their mental or emotional position adopted with respect to their judgements and opinions of the performances in the simulation. A common *stance* was taken on the importance of addressing unsafe practice, especially for clinicians.

*“If there was some safety issue that came out of that session that they were unable to use a piece of equipment safely or they have some misconceptions that were dangerous and would lead to patient harm, they must be addressed before the end of the session because really if you don’t say anything then by omission they feel that that was an okay thing to do.” Interviewee #5*

### Techniques

This category explores techniques used by the interviewees to promote a productive and safe learning environment.

#### Plan of action

This action-oriented theme is in some ways the practical application of the strategizing theme. It has three overlapping subthemes: *structuring*, *methods*, and *sequencing*. The interviewees consistently described a three-phase *structure*: reactions (e.g. learners venting or expressing emotions), discussion (e.g. sharing facts, summarising the scenario, facilitating reflection, seeking understanding, analysing actions) and summary (e.g. identifying take home messages, transfer to clinical practice).

*“There’s a couple of different names for these but it’s the ones that have three phases, which is essentially gathering the information then analysing what actually happened and then basically taking it back to what did they learn from this process and what they can improve upon.” Interviewee #25*

The *structuring* subtheme is about permitting learners to know what is happening and why, and to keep the debriefing on time.

*“Make sure the participants know the structure of the debrief, how it will unfold, make sure they’re aware of their expectations and the rules” Interviewee #10*

The *methods* subtheme captures the many approaches being used within the structure. The interviewees frequently mentioned Plus/Delta [14], Advocacy/Inquiry [16], and Pendleton’s model for feedback [42]. The *sequencing* subtheme refers to the choices made regarding order of events or issues to be discussed during debriefing. Some of these were planned a priori, such as the use of the Plus/Delta [14] as an approach to facilitate prioritisation at the commencement of a debriefing.

#### Creating a safe learning environment

The interviewees described key features in creating a safe learning environment. There was no single approach for how to do this, but the interviewees emphasised establishing ground rules of respect and confidentiality, and setting the learners up for success with scenarios at the appropriate level of challenge. That is:

*“being respectful of people by not purposely trapping them, so you’re actually setting up an area, a zone of safety in training by acknowledging that sometimes challenging things happen and why it’s been done that way.” Interviewee #3*

The interviewees emphasised the importance of briefing before the scenario in which the expectations, rules and structure of the debriefing were outlined. How briefings were delivered varied considerably. Although briefings were mentioned by the interviewees to be essential for creating a safe learning environment, briefing practices are not the focus and have not been analysed independently. Other common techniques for creating a *safe* environment that promoted learning included assisting the participants to de-role after the simulation; setting the stage for the debriefing; acknowledging feelings of the participants; normalising incidents; and sharing responsibility.

#### Managing learning objectives

The role of learning objectives was discussed at length. The interviewees described how learning objectives were of major importance when designing a course or scenario in order to know what action to take, what to look for and when to terminate a scenario. Standardised courses like Anaesthetic Crisis Resource Management (ACRM), Advanced Life Support (ALS) and many student curricula require that prescribed learning objectives should be met. The interviewees had to balance these requirements with their core philosophy of *learner-centredness*:

*“I think the learning objectives are important to determine the course of the scenario and to know when you met what you wanted to do with the scenario. However, I sometimes think that what you planned to happen in the scenarios didn’t always happen, it can be really quite dynamic.” Interviewee #3*

Managing this tension requires the artistry of *thinking on your feet*:

*“…you need to be flexible enough to actually deal with the learner's needs rather than the rigid learning objectives for that scenario” Interviewee #11*

Likewise, the interviewees described prioritisation through confining the number of learning objectives, generally two to four, addressing different facets of practice depending on the learner group and the topics. There was a strong sense that too many objectives and learning was diminished.

*“You can’t get through all the things that you pick up you’ve just got to do the important things and you can’t cover them all” Interviewee #14*

#### Promoting learner reflection

Most of the interviewees believed that the debriefing is just the beginning of the reflective process intended to promote, enable and support the learners’ reflection and learning.

*“I think the whole point really is that we’re getting them to review and reflect upon their practice” Interviewee #21*

Some of the interviewees use the pause and discuss technique [43] to encourage and train reflection-in-action [44] with a notion of the potential transfer of this ability to clinical encounters. This technique was mostly used with learners who had little or no clinical experience. A technique used across all levels was outlining lessons learned with a view to enacting learning in clinical practice.

#### Co-debriefing

Co-debriefing was valued but not a common practice, as it was often constrained by logistics and cost. Benefits included offering of content expertise, especially when content experts lacked debriefing experience. Co-debriefing sometimes afforded added attention to individual learners and also for co-debriefers facilitated peer support, especially when training new debriefers.

*“it’s also just useful to have two brains; you have two people watching the reactions and can redirect questions and can pick up on things you forget.” Interviewee #13*

*“Part of our debriefer mentoring program is that, particularly debriefers who are learning get mentored and get feedback after each debrief from their co-debriefer who is their mentor.” Interviewee #11*

### Development

This category captures how the interviewees’ changed and developed their debriefing practice over time. A key part of development is reflecting on ways to improve debriefing and then enacting them.

#### Transformation

The interviewees were generally aware of the transformations they had made to their debriefing practice over time. In particular, they noted an increased focus on the learners’ need:

*“The main way it’s changed is recognising the fact that I couldn’t apply the same emphasis of debriefing phases to every group, that it needed to change all the time and identify which groups would need more of which aspect of debriefing and which part I’d focus on” Interviewee #25*

#### Challenges

Challenges described by the interviewees can be grouped into two categories: (1) personal and (2) organisational. The personal challenges were the most prominent. The interviewees found it to be a challenge, albeit one often with positive outcomes, to keep on top of new debriefing techniques and methods:

*“Keeping on top of new techniques and ways of doing things and not becoming stayed in what you do. Just having the opportunity to discuss it with others and continue to learn from peers is important” Interviewee #2*

The interviewees reported being challenged by lack of time to do simulation and to debrief; a few interviewees mentioned that debriefing participants with a different clinical background is sometimes challenging.

*“On a interpersonal level I occasionally find that being a nurse debriefing doctors I have to prove my value so that they will listen.” Interviewee #19*

#### Becoming comfortable with the uncomfortable

Through experience and experiment, most of the interviewees reported becoming more comfortable with the learners’ reactions to tricky situations and not knowing where the debriefing is going. The interviewees were aware of uncomfortable situations, and they use these as learning opportunities. In the process, they gained confidence and became relatively comfortable even in unexpected, challenging and uncomfortable situations.

*“Accept that we will never become fully comfortable with the whole process no matter how many years’ experience you get” Interviewee #24*

*“Fundamentally my role is not to be their friend but that my role is to make them think about things and stimulate them to be reflective about their own practice” Interviewee #9*

#### Care about their practice

Some of the interviewees expressed pride in being part of the simulation community and were humble about their own debriefing abilities. They expressed a continued interest to expand their knowledge and debriefing style. Curious to explore different approaches, the interviewees attended courses and workshops among other approaches:

*“I'm of the belief if you ripe you rot very quickly so I think that you continuously need to improve and I think that the way to do that is by cross fertilising or going to other units and spending some time with them and adopting some of their methods and some of their ways on how to debrief” Interviewee #1*

#### Learning from and with peers

The interviewees were deliberate in their use of peer feedback to improve and strengthen current debriefing practice, especially at simulation centres with a larger faculty. Those without peer faculty present for feedback have established peer relationships through the broader simulation community. This includes seeking feedback on recorded debriefings from geographically distant peers. The interviewees use observation of others’ debriefings as inspiration and positive or negative modelling. Peer feedback and observation of others performing debriefing were important pathways to gaining expertise.

*“I learnt an enormous amount having the opportunity to practise my debriefing in front of my colleagues, even though I found it quite threatening initially” Interviewee #2*

*“I think the most constructive and important feedback that we have is from our colleagues” Interviewee #5*

#### Training

Many interviewees reported a rough start with debriefing, as isolated practitioners. They had experience of formal courses and self-study but for many, this came later in their development, and was secondary to the role of peer mentors.

## Discussion

Overall, the peer-nominated experts were remarkably similar in their debriefing structures, using a flexible and blended debriefing approach to support learning after immersive manikin-based simulations. The interviewees expressed **values** or stable fundamental principles that form the foundation of their practices. Descriptions of debriefing were dynamic, creative and individualised, as captured by the category **artistry**. The **technique** category describes the practical approaches and microskills of debriefing, which were broadly convergent. Interviewees also outlined their **development**: how they changed and transformed their debriefing practice over time and learnt to become comfortable in situations that may be otherwise uncomfortable to them or to the learners.

While debriefing approaches across contexts, disciplines and levels of learners’ experience varied, the structure of the debriefing remained consistent and usually followed three phases: reactions, discussion and summarising. This is consistent with the literature [14, 16, 45–47]. The interviewees generally strove to make debriefings learner centred by being honest, revealing their stance and frames, creating a *safe* learning environment, and promoting reflection with a transfer of learning to clinical practice. This also aligns with literature [16, 48–51]. An important aspect of creating a safe learning environment and the ability to maintain it, is directly related to the briefing, which is consistent with what has been described by Rudolph et al. [26]. What this study reveals is the key element of **artistry**, as the interviewees described their comfort with moving flexibly and dynamically between a range of different debriefing models. It also points to the key role of continual development required to become and be an expert debriefer.

### A model for debriefing practice

**Fig. 1:**
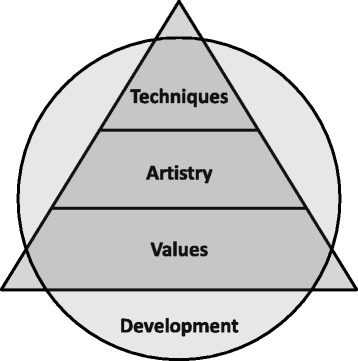
The practice development triangle framework for expert debriefing practices adapted from Handal and Lauvas [52]

To summarise our findings on the debriefing practices of experts, we propose the four categories inform a new model for debriefer development: the practice development triangle (Fig. 1: The practice development triangle framework for expert debriefing practices adapted from Handal and Lauvas [52]). The model is adapted from the theoreticians Handal and Lauvas’ description of educational practice, as it is perceived by the learners [52]. Handal and Lauvas made a distinction between reflections at the three levels of abstraction: (1) practical and manifest action, (2) conceptual level of planning and reflections and (3) ethical considerations, with the “practice triangle” integrating the three levels of practice [52].

We propose that **values** aligns with “ethical considerations” as this is the core philosophical foundation of debriefing; that **artistry** aligns with the conceptual notion of planning and reflections in considering how we are preparing, thinking, wondering and reflecting about how to do it; and that **techniques** aligns with the notion of practical and manifest action. Handal and Lauvas found that the level of actions and techniques may be supported by the two other levels. The findings in our study equally suggest that **values** and ethical consideration are the foundation for **artistry**, planning and reflections, followed by the **techniques** and actions. We have added a surrounding circle of **development** that incorporates, draws from, and informs **values**, **artistry** and **techniques**. This implies that the continuous development is influenced by and will equally influence practice on all the three levels in the practice development triangle.

### Developing debriefers

The practice development triangle provides insight into how we might seek to develop expert debriefers. Often teaching about debriefing focuses heavily on enhancing practical microskills [53, 54]. However, the findings of this study suggest that conceptualising values and developing artistry are at least equally important.

The interviewees relied heavily on observation of peers and peer feedback. This points to the value of structured peer feedback tools like objective structured assessment of debriefing (OSAD) [55] and debriefing assessment for simulation in healthcare (DASH) [56], but even more strongly underlines the need for mentorship. The rise of debriefing courses and graduate programmes in simulation is a relatively new phenomenon [30, 53] and while many of the expert debriefers had undertaken formal training, this was not available at the commencement of their careers. In either case, it is worth reflecting on the possibility that developing **artistry** may be best done relationally through mentorship rather than through a reductionist or theoretical approach.

It is interesting to note that one of the common core values, either implicitly or explicitly described, is being learner centred. In general, interviewees met the needs of their learners by facilitating supportive, constructive, challenging, and reflective discussions through *thinking on their feet* and other forms of **artistry**. This facilitation skill aligned strongly with the notion of *being comfortable with discomfort* (Interviewee #9) and debriefers having to work and practise on the edge of or outside their comfort zone to gain the needed experience. This also has implications for faculty development in debriefing, which are worth considering in further research. That is, when and how to allow faculty to take the risk of going beyond their comfort zone. Learning theories may be most useful to prompt this type extension. We noted that debriefers were highly reflective regarding improving their practical skills, but had reflected less on the conceptual foundations of their debriefing practice. Learning theories provides a means to both validate and challenge simulation practice [57], and we would regard this as an obvious next step in the advancement of debriefing practice.

### Strengths and limitations of the study

The breadth of the national sample across a variety of work contexts is an important feature of this study, and the convergence of the data also supports the strength of the findings. However, although the respondents were nominated as expert debriefers, we have no objective measure of their expertise.

The academic, simulation modality and associated debriefing experiences of the research team may have introduced biases during analysis. However, our differences may also have offered a counterbalance and strengthened our analytic moves. We sought to establish *trustworthiness* through the following strategies outlined by Shenton [58]: purposive sampling, promoting honest responses from interviewees, iterative questioning, regular “debriefing” meetings of research team, our reflective commentary, audit trail and peer scrutiny of the research project. There are limitations to an interview study, as we relied on self-report of practice and we do not know the impact of these practices on the experience of the learners.

Qualitative studies like this one are dependent on the researchers’ approach and preconceptions when data is extracted and interpreted. As with all other interpretive qualitative research, the results are not reproducible or generalizable in a quantitative sense, but the commonalities across our broad sample suggest the findings may have relevance in other SBE contexts.

## Conclusions

This study has described the practices of experts who debrief learners after immersive manikin-based simulations. The findings are applied and presented in a practice development triangle, where the microskills of **techniques** are only made possible through **artistry**, which itself is firmly grounded in the debriefers’ **values**. A key part of the framework is the continuing efforts of debriefers to improve their own practice. The **values** of debriefers provide a foundation for enacting **techniques** and **artistry**. This shift in orientation may offer guidance in designing courses and programmes to support the **development** of debriefing after immersive manikin-based simulations.

## Appendix

### Effective debriefing approaches in simulation based education - Topic guide for semi-structured interviews

Intro

We want to understand from your perspective how and why you debrief as you do.

1. Before we get started on debriefing can you talk a little about your background? …

- *Probe for clinical discipline, professional qualifications, educational qualifications, research experience, training in debriefing, length of time they have been debriefing, frequency of debriefing, context of debriefing etc. Finally ask about age or date of birth*

Now for debriefing specifically.

How you debrief

2. Can you describe the usual contexts in which you debrief after simulations?

- *Probes: Making sure that debriefing describing/discussed is manikin based immersive simulation.*

3. Please describe how you 'usual' run a debriefing session.

- *Probes: Some experts use specific models, or components of them, is this something you do? what models to you use and what advantages/disadvantages are there to this approach*

4. Please describe a powerful debriefing session that you have conducted. What happened and why was it valuable/powerful?

Learners during debrief

5. During debriefing, how do you involve the learner/s?
6. How important are learning objectives in your debriefing?
7. How important is video review in your debriefing sessions?
8. How important is gaining participants’ reaction in your debriefing?
9. Who do you think does most of the talking in your debriefing sessions?
10. What do you think your learners value in a debriefing session?
11. How important is it to offer expert opinion in your debriefing?
12. To what extent do you get feedback from your learners on your debriefing? How? When? What's usual?
13. To what extent do you get feedback from your peers on your debriefing? How? When? What's usual?

Becoming an expert

14. Your peers consider you an expert in debriefing. How do you build this expertise?
15. In what ways has your debriefing changed over time?
16. Are there any significant influences on your debriefing practice? …. Events? Courses? Certain people? Role models?
17. What do you think constitutes an ideal debriefing?
18. What educational theories underpin your approach to debriefing?
19. How do you know when things have not gone well?
20. How do you measure an effective debriefing session?
21. What is the hardest thing about becoming expert in debriefing?
22. I notice that you have/have not spoken about briefing. I wonder how important you think this is for debriefing…?

Beyond expert

23. What challenges do you experience in debriefing?
24. What barriers do you experience in debriefing?
25. How do you think you can improve your debriefing?

Closure

Finishing the interview—what advice will you offer upcoming debriefers?

Although you were nominated as an expert debriefer, I wonder if you can nominate other debriefers? Especially someone who debriefs in a different style/approach to you?

Thanks

Interviewer to make a short recap on the key issues as they have heard them, seek clarification

## Abbreviation

SBE: simulation-based education
